# Research progress and molecular mechanism of oridonin in the treatment of malignant melanoma

**DOI:** 10.3389/fonc.2025.1606325

**Published:** 2025-06-17

**Authors:** Tinghan Deng, Jingping Wu, Hongbin Cheng, Jun Lu

**Affiliations:** ^1^ School of Clinical Medicine, Chengdu University of Traditional Chinese Medicine, Chengdu, China; ^2^ Department of Medical Cosmetology, Hospital of Chengdu University of Traditional Chinese Medicine, Chengdu, China; ^3^ Department of Dermatology, the Third Affiliated Hospital of Chengdu University of Traditional Chinese Medicine, Chengdu, China; ^4^ State Key Laboratory of Southwestern Chinese Medicine Resources, School of Pharmacy, Chengdu University of Traditional Chinese Medicine, Chengdu, China

**Keywords:** oridonin, malignant melanoma, anti-tumor mechanisms, apoptosis, angiogenesis, immune regulation

## Abstract

Malignant Melanoma (MM) is a highly invasive and easily metastasizing skin cancer. Although current treatments have made certain progress in targeted therapy and immunotherapy, drug resistance and side effects remain urgent problems to be addressed. Oridonin, an active diterpenoid compound derived from the traditional Chinese medicine herb *Rabdosia rubescens*, has garnered widespread attention in recent years for its multi-target anti-tumor effects. This review systematically summarizes the research progress of oridonin in the treatment of malignant melanoma, focusing on its multiple molecular mechanisms, including inhibition of tumor cell proliferation, induction of apoptosis, inhibition of invasion and metastasis, suppression of angiogenesis, and modulation of the immune microenvironment. Through *in vitro* cell experiments and *in vivo* animal model studies, oridonin has demonstrated significant anti-melanoma activity and has shown potential synergistic effects when used in combination with existing therapies. Additionally, the optimization of pharmacokinetics and toxicology of oridonin has laid a foundation for its clinical application. However, clinical trial data on oridonin are still limited, and future high-quality clinical studies are needed to verify its safety and efficacy. As a natural product with multiple anti-tumor mechanisms, oridonin exhibits broad prospects as a potential therapeutic agent for malignant melanoma, but further basic and clinical research is required to promote its clinical translation.

## 1 Introduction

Malignant Melanoma (MM) is a highly malignant tumor originating from melanocytes that mainly occurs on the skin but can also occur in the mucous membrane and eye (1). In recent years, with the increase of ultraviolet exposure rate and people’s attention to skin health, the incidence of malignant melanoma has shown an increasing trend (2). Despite its relative rarity, malignant melanoma has a significantly higher fatality rate than other types of skin cancer due to its highly aggressive and rapidly metastatic nature. According to statistics, the five-year survival rate of advanced malignant melanoma is still low, and there is an urgent need to develop new effective treatment strategies (3, 4).

At present, the treatment of malignant melanoma mainly depends on surgical resection, radiotherapy, chemotherapy, targeted therapy, and immunotherapy (5). In recent years, with the progress of molecular biology and immunology, targeted therapies (such as BRAF inhibitors, MEK inhibitors) and immune checkpoint inhibitors (such as anti-PD-1 and anti-CTLA-4 antibodies) have achieved certain results in prolonging the survival of patients (6). However, these therapies still face challenges such as drug resistance, high treatment costs, and significant side effects, which limit their wide application and further improvement of clinical effectiveness (7). Therefore, the development of new, effective, and less toxic anti-melanoma drugs has become the focus of current research.

Oridonin is one of the main active components extracted from *Rabdosia rubescens*, a diterpenoid compound (8). In recent years, oridonin has attracted wide attention due to its various biological activities, especially its remarkable effect in the field of anti-tumor (9). According to the previous literature, oridonin can not only inhibit the proliferation and induce apoptosis of various tumor cells, but also inhibit tumor invasion and metastasis, inhibit angiogenesis, and regulate the immune microenvironment of the body through multiple mechanisms of action (10, 11). These multi-target, multi-pathway anti-tumor properties make it a unique advantage in the treatment of complex and highly heterogeneous malignant melanoma (12).

In addition, natural products and their derivatives have always played an important role in the development of anticancer drugs (13). Because of their structural diversity and biological activity, natural products have become an important source for the discovery of novel anticancer drugs (14). As a natural product, oridonin not only has good pharmacological activity, but its relatively low toxic side effects and multiple mechanisms of action provide a theoretical basis and practical possibility for its application in the treatment of malignant melanoma (15).

Based on the above background, this review aims to systematically summarize the research progress of oridonin in the treatment of malignant melanoma, focusing on its various molecular mechanisms and its anti-tumor effects *in vitro* and *in vivo* experiments. At the same time, this paper will also evaluate the potential and challenges of the clinical application of oridonin, and look forward to its future development direction. Through a comprehensive review of the existing research results, we hope to provide a valuable reference for the further research and clinical application of oridonin as a new anti-melanoma drug.

## 2 Overview of malignant melanoma

### 2.1 Pathological features and epidemiology of malignant melanoma

MM originates from melanocytes and mainly occurs on the surface of the skin, but can also occur in mucous membranes (such as mouth, nasal cavity, perianal) and (such as uvea) (16). MM is highly invasive and can quickly metastasize through lymph and blood circulation to key organs such as liver, lung, brain, and bone, seriously threatening the life of patients (2). According to the location of the disease, cell morphology, and clinical manifestations, MM can be divided into superficial spreading type, nodular type, terminal freckle-like type, and malignant freckle-like type, and these subtypes have significant differences in molecular pathological characteristics, clinical progression rate, and prognosis (17, 18). MM shows high molecular heterogeneity. Common driver gene mutations include BRAF (especially BRAF V600E), NRAS, c-KIT, etc. (19). These mutations are distributed differently in different populations and subtypes, which directly affect the sensitivity to targeted drugs and clinical treatment strategies. Although some patients have a significant response to molecular targeting or immunotherapy in the short term, with the extension of treatment time, tumor cells often develop drug resistance through genomic changes, signaling pathway reprogramming, and microenvironment adaptation, leading to relapse (20). Globally, the incidence of MM is increasing year by year, especially in high ultraviolet radiation areas such as Europe, the United States, and Australia and in white groups (21). The main risk factors of MM include excessive exposure to ultraviolet rays (UVB, UVA), a large number of or atypical moles, family history (especially related to CDKN2A, CDK4, and other genetic factors), and immune dysfunction, among which DNA damage and oxidative stress caused by ultraviolet light are considered to be the core mechanisms inducing malignant transformation of melanocytes (22, 23).

### 2.2 Current status and limitations of treatment of malignant melanoma

For early localized lesions, surgical resection is still the most effective radical treatment (24, 25). However, for advanced patients who have developed extensive metastases, surgery often provides only palliative relief (26). Traditional chemotherapy drugs such as dacarbazine and temozolomide are used in the systematic treatment of melanoma (27, 28), but their response rates are low and they are prone to drug resistance (29). Radiation therapy has a low overall sensitivity to melanoma and is only used as an adjunctive or palliative treatment in a few cases (30). With the development of molecular biology, molecular targeted drugs targeting BRAF V600E mutation and MEK pathway, such as vemurafenib, dabrafenib, and trametinib, have made remarkable progress, significantly prolonging the survival of patients (31, 32). However, most patients develop resistance in the short term after the use of targeted drugs, and some patients do not carry targeted genetic mutations (33, 34). In recent years, immune checkpoint inhibitors, such as nivolumab, pembrolizumab, and ipilimumab, represented by PD-1/PD-L1 (35) and CTLA-4 (36), have significantly improved the long-term survival rate of patients with melanoma and have become an important treatment option, especially in advanced or metastatic cases. However, these drugs are expensive and may cause serious immune-related adverse events, which limit their wide application and general improvement of clinical efficacy (37). Whether through traditional chemotherapy or targeted therapy, melanoma cells can acquire resistance through various mechanisms such as rearrangement (38), *de novo* mutations (39), and alternative signaling pathway activation (40).Additionally, immunotherapy faces the challenge of tumor immune evasion (41). In addition, targeted drugs and immune checkpoint inhibitors are often associated with significant side effects, such as rashes, liver toxicity, endocrine disruption, and severe immune responses, which can lead to treatment interruption (42, 43). At the same time, high drug costs also bring a heavy economic burden to patients and the medical system (44, 45). Although biomarkers such as BRAF V600E mutation and PD-L1 expression level have been used to guide clinical medication to some extent, there is still a lack of systematic, reliable, and universal markers to accurately predict individual efficacy and prognosis (46). The highly heterogeneous nature of melanoma makes it difficult for a single marker to cover all patient subtypes (47). Although early diagnosis and treatment can improve the cure rate, the easy metastatic nature of melanoma means patients remain at high risk of recurrence after surgery or systemic treatment, and the five-year survival rate of patients with advanced or metastatic disease is still low, underscoring the need for new comprehensive treatment strategies to extend survival and improve quality of life (48, 49).

### 2.3 New therapeutic strategies and potential value of Chinese medicine components

Multi-target or multi-pathway intervention, or the combination of chemotherapy, targeted therapy, and immunotherapy (50), can help overcome the problem of resistance to single drugs (51, 52). The rise of personalized medicine and precision medicine further requires screening of more matched treatment options at the genetic, protein, and metabolic levels to obtain optimal efficacy and minimize side effects (53). For example (54), by using tyrosinase as a “needle” and dual-functionalized polysaccharides with tyrosine and triphenylphosphine as a “rope,” researchers constructed a melanin network within melanoma cells to target mitochondria and slow down tumor metabolism. Simultaneously, combining this approach with photothermal therapy to inhibit tumor growth offers a novel strategy for non-pharmacological organelle regulation in cancer treatment.

There are abundant anti-tumor active molecules in traditional Chinese medicine and other national medicines, which provide a new possibility for the treatment of melanoma (55, 56). These natural products typically have multiple targeting mechanisms, relatively low toxicity side effects, and a wide range of raw material sources (57). A number of previous studies have shown that the active monomer or compound of traditional Chinese medicine can inhibit the growth and metastasis of malignant melanoma by inducing tumor cell apoptosis, inhibiting angiogenesis, and regulating the tumor immune microenvironment (58). For example, research has shown (59) that the water-soluble total protein extract from *Pueraria* (PLP) can effectively inhibit melanin synthesis and proliferation in B16 melanoma cells by suppressing tyrosinase activity, downregulating MITF and its associated melanogenic enzymes, and triggering apoptosis via the mitochondrial pathway.

Among these natural compounds, oridonin, a bioactive diterpenoid isolated from *Rabdosia rubescens*, has garnered significant attention in cancer research due to its multi-target mechanisms and low toxicity. Current research trends highlight its potential in cancer treatment, particularly in gastric cancer, where it has been shown to induce apoptosis through caspase activation and inhibit the PI3K/AKT/mTOR signaling pathway, which is frequently dysregulated in malignancies (60). However, there is a notable lack of data on oridonin’s efficacy and mechanisms in melanoma, leaving a critical gap in understanding its broader therapeutic applications.

Future studies should focus on exploring oridonin’s role in other cancer types, such as melanoma, to validate its multi-target effects and immune-modulating capabilities. For instance, its ability to enhance the activity of cytotoxic T cells and natural killer (NK) cells, thereby promoting an anti-tumor immune response, could be particularly relevant in cancers with immune evasion mechanisms. Additionally, oridonin’s selective targeting of cancer cells while sparing normal tissues minimizes systemic toxicity, making it a safer alternative to traditional chemotherapeutic agents.

The integration of oridonin into multi-target therapeutic strategies, combined with the synergistic effects and low toxicity profiles of other traditional Chinese medicine components, offers a promising avenue for the development of personalized and precision medicine approaches in cancer treatment. This aligns with the growing emphasis on leveraging natural compounds to address the limitations of conventional therapies, such as drug resistance and adverse effects, and to provide more effective and safer treatment options.

## 3 Basic information and pharmacological study of oridonin

**Figure 1:**
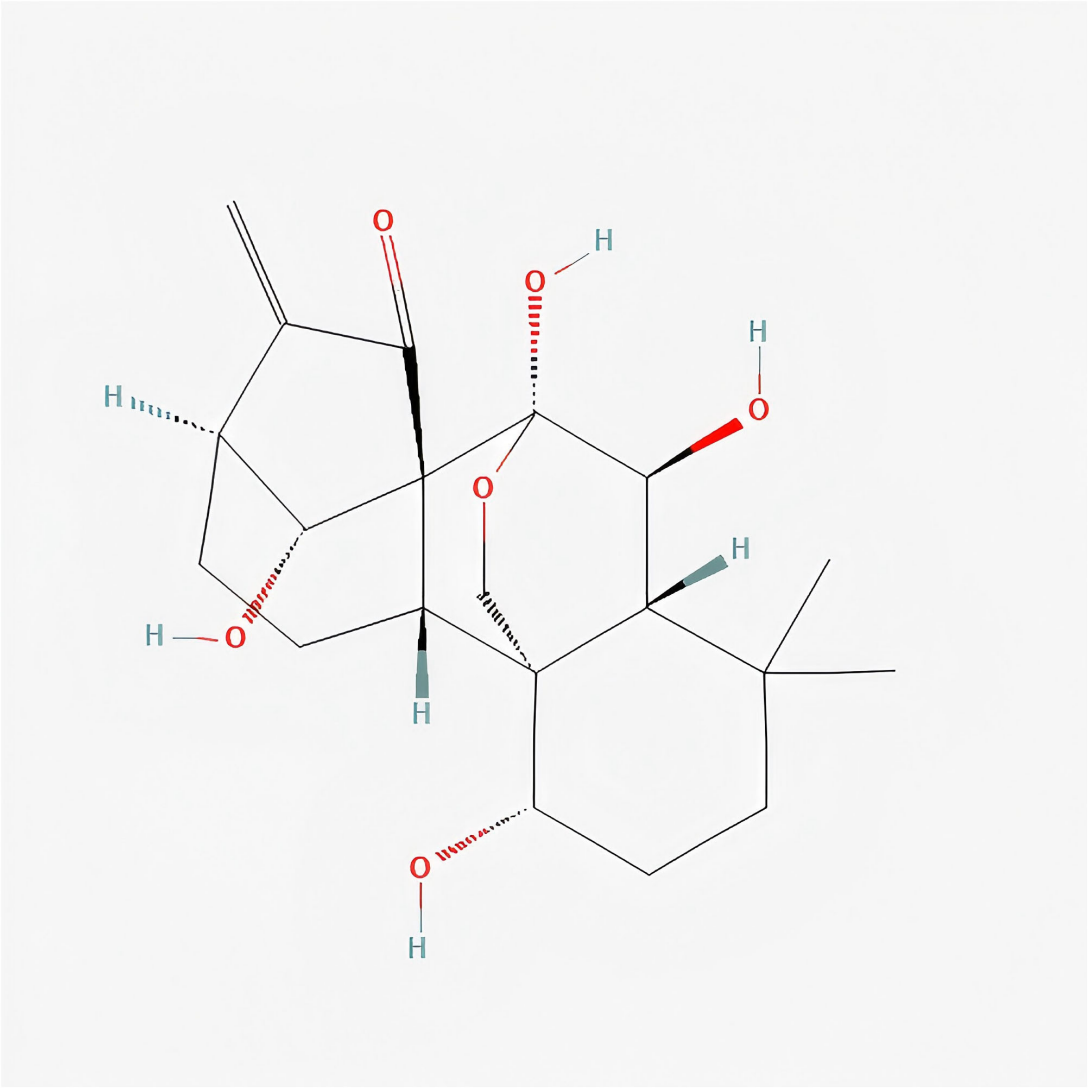
Oridonin’s 2D Structure. The chemical structure of Oridonin in **FIGURE 1** of this manuscript is cited from PubChem (95), and the specific URL is as follows: https://pubchem.ncbi.nlm.nih.gov/compound/5321010.

Oridonin is a diterpenoid compound with the chemical formula C_20_H_28_O_6_, extracted from Rabdosia rubescens, a plant of the Lamiaceae family (61) (See **Figure 1**) (62). It typically exists in the form of needle- or plate-like crystals and possesses a unique core structure: an ent-kaurane-type diterpenoid skeleton (63). Structurally, oridonin features multiple functional groups (including hydroxyl and ketone groups), which often play crucial roles in its binding to cellular targets (9, 64). The pharmacological activities of oridonin are not limited to its anticancer effects but also include the following aspects:

Anti-inflammatory and immunomodulatory: Studies have shown (65) that oridonin mainly exerts its immunomodulatory and anti-inflammatory effects by inhibiting the activation of the NLRP3 inflammasome and reducing the release of inflammatory factors such as IL-1β and IL-18. This makes it a potential candidate compound for the treatment of inflammatory diseases.
Anti-bacterial and anti-viral: Some literature reports (66) indicate that oridonin and its derivatives exhibit inhibitory activity against Gram-positive and Gram-negative bacteria, as well as several viral strains.
Cardiovascular protection: Research has found (67) that oridonin can effectively reduce oxidative stress levels in Atherosclerosis related models and also provides some protection to vascular endothelial function.

However, oridonin is most notably recognized for its potential in cancer therapy, encompassing multiple mechanisms such as inducing tumor cell apoptosis, blocking the cell cycle, inhibiting angiogenesis, and enhancing immune function (68).

## 4 Molecular mechanism of oridonin in inhibiting malignant melanoma

Oridonin is a natural diterpenoid compound extracted from the Labiatae plant *Rabdosia rubescens* and other plants. Due to its novel structure and relatively low toxic side effects, it has attracted much attention in the research of various malignant tumors in recent years (69). In malignant melanoma, oridonin exerts a synergistic anti-tumor effect through multiple signaling pathways and molecular targets, including inducing apoptosis, blocking the cell cycle, inhibiting angiogenesis, suppressing cell migration and invasion, regulating multiple key signaling pathways, reshaping the tumor immune microenvironment, and interfering with autophagy and metabolic reprogramming.

### 4.1 Inducing apoptosis

**Figure 2:**
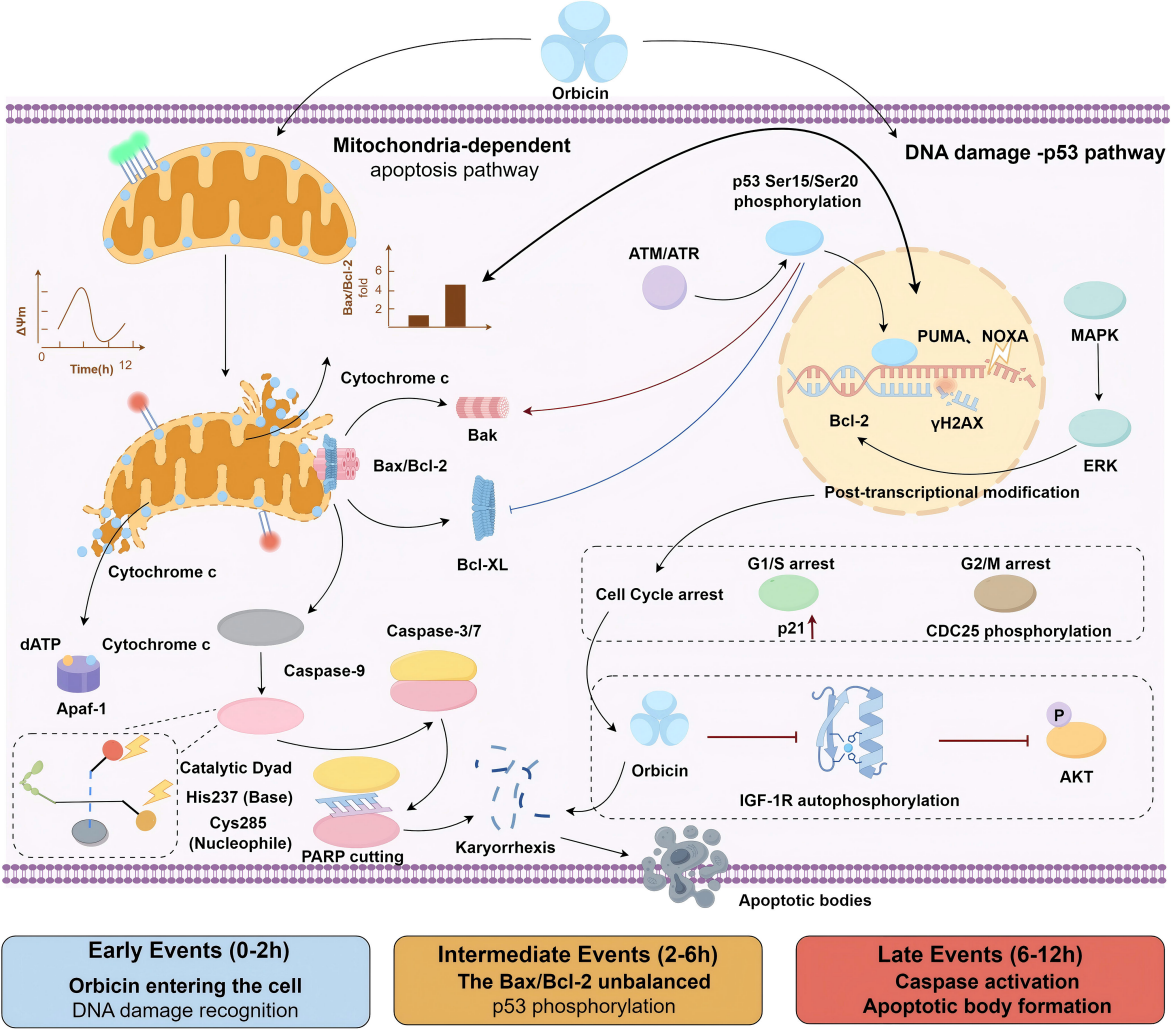
Oridonin-induced apoptosis of MM cells is mainly dependent on mitochondria-dependent apoptosis and activation of p53 pathway related to DNA damage response. **FIGURE 2** in this manuscript was drawn by ourselves, and the drawing was done using the Figdraw website.

The apoptosis of MM cells induced by oridonin mainly depends on the following pathways: mitochondrial-dependent apoptosis and the activation of the p53 pathway related to DNA damage response. The mechanism pathway diagram is shown in **Figure 2**.

#### 4.1.1 Mitochondrial-dependent apoptosis

Zhang CL (70) et al. found that oridonin induces apoptosis in A375-S2 cells through the caspase pathway regulated by Bax, which depends on the cytochrome c/caspase-9 apoptosome. In the intrinsic pathway, oridonin upregulates the expression of pro-apoptotic proteins Bax and Bak, while downregulating the levels of anti-apoptotic proteins Bcl-2 and Bcl-XL, resulting in a significant increase in the Bax/Bcl-2 ratio. This change disrupts the integrity of the outer mitochondrial membrane, leading to the loss of mitochondrial membrane potential and the release of cytochrome c into the cytoplasm. Cytochrome c in the cytoplasm combines with Apaf-1 and dATP to form an apoptosome, which then activates caspase-9, subsequently activating downstream caspase-3 and caspase-7 in a cascade reaction, ultimately triggering programmed cell death. Moreover, some studies have shown (71) that the pro-apoptotic effect of oridonin is partly attributed to its interference with IGF-1R signal transduction, leading to the activation of p53 protein and the initiation of the intrinsic apoptotic pathway in cells.

#### 4.1.2 DNA damage response and p53 pathway

Chun-Ling Zhang (72) et al. found that oridonin can promote apoptosis by activating the p53 and ERK signaling pathways, including regulating the expression ratio of Bax and Bcl-xL proteins and inducing the release of mitochondrial cytochrome c into the cytoplasm. Specifically, oridonin can trigger the DNA damage response pathway in the form of DNA double-strand breaks or base mismatches, activating checkpoint kinases such as ATM/ATR, and stabilizing and activating the p53 protein. The activated p53 not only upregulates the transcription of apoptosis genes such as PUMA and NOXA, promoting cell apoptosis, but also blocks the cell cycle at the G1/S or G2/M phase when the DNA damage is repairable, providing time for repair. Once the repair fails, the p53 signaling axis will further strengthen the apoptotic program, thereby eliminating severely damaged cells.

### 4.2 Inhibiting angiogenesis

Malignant melanoma has a high demand for blood supply. Oridonin weakens the formation of tumor neovascularization through multiple pathways (73). Jin-Huan Jiang et al. (74) found in *in vitro* experiments that oridonin not only inhibits the proliferation, migration, and tube formation of endothelial cells induced by vascular endothelial growth factor (VEGF), but also causes human umbilical vein endothelial cells (HUVECs) to arrest at the G2/M phase and induce apoptosis. Mechanism studies have shown that the anti-angiogenic effect of oridonin is at least partly due to the downregulation of VEGFR2-mediated FAK/MMPs, mTOR/PI3K/Akt, and ERK/p38 signaling pathways, thereby reducing the invasion, migration, and tube formation of HUVECs. In addition, oridonin can down-regulate the gene and protein expression of VEGF and its receptor VEGFR-2 (KDR/Flk-1), interfere with subsequent kinase cascade reactions, and significantly reduce the formation of new blood vessels. By inhibiting the VEGF/VEGFR signaling pathway, oridonin effectively blocks the angiogenic requirements of tumors, thereby inhibiting the growth and spread of malignant melanoma.

### 4.3 Inhibition of cell migration and invasion

Oridonin can significantly weaken the invasiveness of melanoma cells and reduce their metastatic potential. Chun-Yu Li et al. (75) found that oridonin mainly inhibits the migration, invasion, and adhesion of melanoma cells by suppressing the PI3K/Akt/GSK-3β signaling pathway, and simultaneously inhibits the EMT process induced by TGF-β1. In terms of EMT reversal, oridonin can down-regulate the expression of key transcription factors such as Snail, Slug, Twist, and ZEB1, reduce the content of N-cadherin and vimentin, and up-regulate E-cadherin, thereby maintaining the epithelial phenotype of cells and blocking their transformation into a highly invasive state.

### 4.4 Regulation of the immune microenvironment

Malignant melanoma employs multifaceted strategies to evade immune surveillance, including upregulation of the PD-L1/PD-1 axis, where tumor cell-derived PD-L1 binds PD-1 on T cells to suppress their activation and effector functions. Concurrently, the tumor microenvironment (TME) becomes immunosuppressive through infiltration of regulatory T cells (Tregs) and myeloid-derived suppressor cells (MDSCs), which secrete cytokines like IL-10 and TGF-β to establish a tolerogenic niche. Additionally, melanoma cells impair antigen presentation by downregulating MHC-I expression and disrupting antigen-processing machinery, thereby escaping detection by CD8+ T cells (76). Oridonin promotes the anti-tumor activity of immune cells through multiple mechanisms, thereby reversing this escape phenomenon (77). In contrast, the bioactive diterpenoid oridonin counteracts these immune evasion mechanisms through multimodal immunomodulation. It enhances cytotoxic lymphocyte activity by amplifying tumor-infiltrating CD8+ T and natural killer (NK) cell populations, upregulating their production of perforin, granzyme B, and interferon-γ (IFN-γ), which collectively enhance melanoma cell recognition and lysis.According to the studies by Guo et al. and Hwang et al., oridonin regulates different immune cell signaling pathways, demonstrating its immunomodulatory and antitumor effects. In the study by Guo et al., oridonin inhibits the TGF-β receptor signaling pathway by reducing the phosphorylation of downstream Smad2/3, thereby suppressing the differentiation of regulatory T cells (Tregs) and enhancing antitumor immune responses. Key experimental parameters include the use of 4T1 breast cancer cell lines and BALB/c mouse models, oridonin concentration gradients of 5 μM and 10 μM, flow cytometry to detect the proportion of Tregs, Western blot to analyze changes in signaling proteins, and ELISA to measure TGF-β levels. In the study by Hwang et al., oridonin enhances the cytotoxic activity of natural killer (NK) cells, potentially by activating the NKG2D receptor and promoting the release of perforin and granzyme. Key experimental parameters include the use of A549 lung cancer cell lines and human peripheral blood NK cells, oridonin concentration gradients of 10 μM and 20 μM, CCK-8 assays to evaluate tumor cell viability, LDH release assays to assess cytotoxicity, flow cytometry to detect NK cell activation markers, and ELISA to measure cytotoxic factors. These two studies reveal that oridonin exerts its antitumor effects by modulating the TGF-β receptor signaling pathway to inhibit Treg differentiation and by enhancing NK cell activity through the activation of cytotoxic pathways (78, 79). These synergistic actions position oridonin as a promising candidate for reversing melanoma-induced immune tolerance. Yan-Feng Liu et al. (80) found that oridonin can significantly increase the killing efficiency of NK-92 MI cells on THP1 cells, and this effect may be closely related to its up-regulation of the expression levels of NKG2D ligands such as MICB, ULBP1, and ULBP2, as well as the promotion of the release of interferon-γ and tumor necrosis factor-β. However, the current relevant research evidence is still limited, and the experiments were not specifically conducted for malignant melanoma but were widely verified *in vitro* with various cancer cells. Further experimental verification is needed to establish its specific mechanism and clinical application potential in MM.

*In vitro* experiments serve as the foundation for evaluating the anti-malignant melanoma activity of oridonin. Through the aforementioned studies, it is evident that various melanoma cell lines (such as A375) are utilized to systematically assess its effects on tumor cell proliferation, apoptosis, migration, and invasion capabilities. Firstly, MTT or CCK-8 cell proliferation assays demonstrate that oridonin significantly inhibits the proliferation of MM cells in a dose-dependent manner. For instance, in A375 cells, the IC50 value of oridonin is approximately 5-15 μM, and cell viability significantly decreases with increasing doses (60). Additionally, colony formation assays further confirm the inhibitory effect of oridonin on MM cell proliferation, as the number of cell colonies is markedly reduced after treatment, indicating its potential for long-term suppression of cell proliferation (75). In apoptosis studies, flow cytometry analysis shows that the apoptosis rate of MM cells significantly increases after treatment with oridonin (70). Further Western blot analysis reveals that oridonin upregulates the expression of pro-apoptotic proteins such as Bax and caspase-3 while downregulating the expression of anti-apoptotic protein Bcl-2, suggesting that it induces cell apoptosis by modulating the Bcl-2 family proteins and the caspase pathway (70, 72). Furthermore, Hoechst 33342 staining and Annexin V-FITC/PI double staining assays also show that MM cells treated with oridonin exhibit typical apoptotic morphology, such as nuclear fragmentation and chromatin condensation (70). The effects of oridonin on the migration and invasion capabilities of MM cells are assessed through scratch assays and Transwell invasion assays. The results indicate that oridonin significantly inhibits the migration and invasion of MM cells, with the inhibitory effect positively correlated with the dose (75). Molecular mechanism studies further reveal that oridonin downregulates the expression of matrix metalloproteinases (MMP-2, MMP-9) and inhibits the expression of epithelial-mesenchymal transition (EMT)-related transcription factors such as Snail and Twist, thereby reducing the invasive and migratory potential of MM cells (75).

### 4.5 Tumor-specific targeting and minimized off-toxicity

Oridonin, a natural antitumor compound, achieves tumor-specific targeting and reduces off-target effects through synergistic strategies combining targeted delivery systems and selective cytotoxicity. Current studies demonstrate that drug targeting specificity can be enhanced by nanocarriers to improve tumor tissue accumulation while minimizing systemic toxicity (81). Furthermore, chemical modifications, such as biotin conjugation or integration of redox-active moieties, enhance tumor cell selectivity and induce specific cell death pathways (82, 83).

The application of nanocarrier systems represents a primary strategy to enhance tumor targeting. Nanoparticles leverage the enhanced permeability and retention (EPR) effect in tumor tissues for selective drug delivery, thereby reducing off-target effects (81). For instance, biotin-conjugated gold complexes disrupt redox homeostasis in tumor cells by inhibiting thioredoxin reductase (TrxR) and inducing ferroptosis, further improving tumor selectivity (82).

Studies on nanocarriers reveal that nanoparticle-mediated drug delivery significantly increases drug accumulation in tumor tissues while sparing normal cells. For example, engineered nanoparticles improve drug permeability and tumor tissue retention, a strategy widely applied in treating breast cancer, lung cancer, and other solid tumors (81). This approach provides a promising technical foundation for targeted delivery of oridonin. For instance, for the melanoma subtype with overexpression of FR+ and CD44, the dual-targeting nanoparticles (folic acid-hyaluronic acid conjugates) provide a new approach for the targeted delivery of podophyllotoxin through a GSH-responsive release strategy (84). Similarly, aptamer-functionalized drugs (such as triptolide) enhance tumor-specific targeting through a controlled release mechanism, suggesting that the combination of oridonin with aptamers may improve its delivery efficiency (85).

Additionally, biotin-conjugated gold complexes exhibit remarkable tumor cell selectivity. By suppressing TrxR activity and triggering ferroptosis, these complexes enhance radiosensitivity in tumor cells with minimal impact on normal tissues (82). This mechanism offers critical insights into optimizing oridonin’s tumor selectivity through tailored chemical modifications.

Redox-active compounds, such as the M/A drug combination, also demonstrate tumor-specific cytotoxicity. These agents induce mitochondrial superoxide overproduction and membrane depolarization in tumor cells, achieving selective cytotoxicity while preserving normal cell viability (83). This redox modulation mechanism suggests that oridonin could exploit similar pathways for tumor-specific targeting.

Current trends in antitumor drug targeting focus on optimizing delivery systems and chemical modifications. Nanocarrier technology has advanced to clinical trials, demonstrating significant advantages in multiple cancer types (81). Furthermore, emerging strategies targeting tumor-specific vulnerabilities, such as redox homeostasis disruption and ferroptosis induction, provide novel directions for drug design (82, 83).

However, existing studies face limitations, including insufficient long-term safety assessments of nanoparticles and incomplete understanding of their behavior in complex tumor microenvironments. Future research should integrate oridonin’s structural features with nanocarrier delivery and chemical modifications to enhance targeting specificity, therapeutic efficacy, and systemic safety.

## 5 Clinical translation and application prospects of oridonin in malignant melanoma

Oridonin, a natural diterpenoid compound with multifaceted anti-tumor activity, has shown significant promise in the treatment of MM in recent years. However, the transition from basic research to clinical application presents numerous challenges and opportunities.

### 5.1 Preclinical studies

Comprehensive preclinical studies are essential for the clinical translation of oridonin. Existing research demonstrates that oridonin exerts significant inhibitory effects on MM cells both *in vitro* and *in vivo*. For instance, *in vitro* studies using A375 melanoma cell lines have shown that oridonin induces apoptosis, suppresses angiogenesis, and inhibits cell migration and invasion, effectively reducing tumor growth and metastasis (60, 75). *In vivo* studies in xenograft mouse models further confirm these findings, with oridonin significantly reducing tumor volume and metastatic potential (70). However, preclinical research remains insufficient. Beyond validating its anti-tumor efficacy, further investigation into its pharmacokinetics, optimal administration routes, and dose optimization is essential to establish a solid scientific foundation for subsequent clinical trials.

### 5.2 Safety and toxicity evaluation

The clinical translation of oridonin necessitates thorough evaluation of its safety and toxicity. Although *in vitro* and animal studies suggest relatively low toxicity, its safety profile in humans must be rigorously validated through clinical trials. For example, in a preclinical toxicity study, oridonin exhibited a favorable safety profile in mice, with no significant organ toxicity observed at therapeutic doses (60). However, determining the maximum tolerated dose (MTD) and safe therapeutic window is a critical prerequisite for its clinical application. Additionally, the long-term safety of oridonin and its impact on normal cells warrant in-depth investigation to ensure its clinical use is both safe and sustainable.

### 5.3 Pharmacokinetics and pharmacodynamics

Pharmacokinetic (PK) and pharmacodynamic (PD) studies are pivotal for the clinical application of oridonin. Existing evidence suggests that the absorption, distribution, metabolism, and excretion (ADME) characteristics of oridonin *in vivo* are complex, which impacts its bioavailability and anti-tumor efficacy (9). For instance, studies have shown that oridonin has low oral bioavailability due to extensive first-pass metabolism, necessitating alternative administration routes such as intravenous delivery (86). To optimize its clinical use, advanced pharmacokinetic modeling and pharmacodynamic evaluation are required to determine the optimal route of administration (e.g., oral or intravenous), dosing frequency, and dose intensity. Additionally, the application of nanotechnology and drug delivery systems—such as liposomal encapsulation and nanoparticle carriers—has been shown to significantly enhance the bioavailability and targeting efficiency of oridonin, while reducing systemic toxicity and improving therapeutic efficacy (86, 87).The design of hydroxyl acid-sensitive acetal ester bonds (88) indicates that the targeted release of drugs in the tumor microenvironment can significantly enhance therapeutic efficacy. Such technology may be applied to the development of nanoformulations of oridonin to optimize its pharmacokinetic properties.

### 5.4 Combination therapy strategies

Given the high heterogeneity and complex resistance mechanisms of malignant melanoma, monotherapy often fails to achieve satisfactory outcomes. Oridonin, with its multi-target and multi-mechanism mode of action, holds potential for combination therapy with other treatment modalities, such as targeted therapy, immunotherapy, radiotherapy, and chemotherapy (89). In addition to malignant melanoma, other types of malignant tumors have been extensively studied in terms of combination therapy strategies. For example, research has shown (90) that the combination of oridonin with BRAF inhibitors can effectively delay the development of resistance. When combined with immune checkpoint inhibitors (78), it may further enhance antitumor effects by boosting the host immune response. Firstly, the role of the PI3K/AKT signaling pathway in tumor drug resistance has been widely discussed. The excessive activation of this pathway promotes the proliferation, invasion and metastasis of tumor cells by regulating downstream proteins, while resisting conventional treatment (91). The inhibitory strategy targeting the PI3K/AKT pathway is regarded as an important direction to overcome drug resistance, which may provide a reference for the research of oridonin. Secondly, the adaptive changes of mitochondria in tumor drug resistance are another important mechanism. Mitochondria provide energy and metabolic flexibility for tumor cells through dynamic changes and signal integration, thereby enhancing their viability (92). These adaptive changes include mitochondrial dynamics regulation, metabolic pathway remodeling, and mitochondrial-mediated apoptosis regulation. (92) points out that drug development and delivery strategies targeting mitochondria may effectively overcome chemotherapeutic resistance. Furthermore, other studies have also mentioned the role of tumor stem cells (such as breast cancer stem cells) in drug resistance. These cells enhance their resistance to radiotherapy and chemotherapy through complex signaling pathway reprogramming and interaction with the tumor microenvironment (93, 94). Although these mechanisms do not directly mention melanoma, similar molecular mechanisms may also apply to the drug resistance of melanoma. These explorations of combination therapy strategies provide diverse options for the clinical application of oridonin, which holds significant clinical importance.

## 6 Discussion

In the context of current therapeutic strategies for melanoma, overcoming tumor heterogeneity and immune evasion remains a primary challenge in both clinical and research domains. On one hand, common genetic mutations in melanoma, such as BRAF, NRAS, and NF1, result in multiple potential resistance pathways within the MAPK signaling axis. Although BRAF or MEK inhibitors initially achieve promising therapeutic outcomes, resistance inevitably emerges rapidly (40). On the other hand, the tumor immune microenvironment (TIME), characterized by the infiltration of immunosuppressive cells (e.g., Tregs and MDSCs) and the overexpression of immune checkpoint molecules, impairs the host immune system’s ability to generate durable and effective cytotoxic responses against melanoma cells (45). While the advent of immunotherapy has brought new hope to patients with advanced melanoma, not all individuals respond to immune checkpoint inhibitors (ICIs) targeting PD-1/PD-L1 or CTLA-4, with primary or acquired resistance being a persistent obstacle in a subset of patients (36).

Against this backdrop, oridonin, through its multi-target and multi-mechanism modes of action, exhibits significant potential for combating melanoma. It not only directly inhibits tumor cell proliferation and invasion but also interferes with angiogenesis and enhances the cytotoxic activity of immune cells. These properties provide a rationale for its use in combination therapy strategies. For example, combining oridonin with BRAF or MEK inhibitors could simultaneously target escape routes within the MAPK signaling axis and alternative proliferation pathways, thereby delaying or potentially reversing resistance (90). Additionally, its immunomodulatory effects on the tumor microenvironment (TME) establish a theoretical foundation for combination with PD-1/PD-L1 or CTLA-4 inhibitors, potentially overcoming immune tolerance in certain patients (78). Notably, as emerging immune checkpoints such as LAG-3 and TIM-3 are integrated into clinical trials, and engineered T-cell therapies (e.g., CAR-T or TCR-T) are expanded into solid tumors (91, 95), oridonin’s potential to activate both innate and adaptive immunity may enable synergistic effects with these novel immunotherapeutic strategies.

However, for oridonin to transition from bench to bedside, several critical challenges must be addressed. First, it’s *in vivo* pharmacokinetics (PK) remain poorly understood. Factors such as low bioavailability, variability in plasma concentrations across different administration routes (e.g., oral vs. intravenous), and limitations imposed by first-pass hepatic metabolism and plasma protein binding need to be carefully studied (86). Second, there is a lack of systematic studies on the optimal dosage range, interactions with existing first- or second-line therapies, and potential toxicity to normal tissues (87). To address these limitations, the development of advanced drug delivery systems—such as nanoparticle formulations and liposomal encapsulation—may improve oridonin’s stability, targeting specificity, and therapeutic index, while minimizing toxicity. Such innovations could pave the way for large-scale clinical trials (86, 87). Third, given the high heterogeneity in melanoma’s molecular subtypes and disease stages, patients’ responses to oridonin—both in terms of pathway regulation and immune remodeling—are likely to vary. This underscores the importance of identifying biomarkers that can guide patient selection and align therapeutic strategies within the framework of precision medicine (89).

Oridonin demonstrates multiple mechanisms of action that align closely with current trends in molecularly targeted therapy and immunotherapy for melanoma. It also has the potential to address some of the limitations and challenges associated with monotherapies. However, existing research remains insufficient, and robust supporting data are urgently needed. From both research and clinical perspectives, future efforts should focus on comprehensive investigations into its pharmacokinetics, toxicology, and efficacy in large animal models. Early-phase, multicenter clinical trials should then explore optimal dosing regimens and combination therapy strategies. Simultaneously, leveraging molecular subtyping and biomarker-driven precision medicine approaches could help identify patient populations most likely to benefit from oridonin-based therapies. Achieving these breakthroughs is essential for oridonin to emerge as a safe and effective “novel weapon” in the fight against melanoma, addressing the clinical challenges of treatment resistance, recurrence, and disease progression.

## 7 Conclusion

Oridonin, a natural diterpenoid compound extracted from *Rabdosia rubescens* and other plants of the Lamiaceae family, has demonstrated significant anti-tumor potential in preclinical studies and basic research on malignant melanoma. Existing literature and experimental findings indicate that oridonin exerts its anti-cancer effects through multi-target and multi-pathway mechanisms, primarily including the induction of intrinsic apoptosis, inhibition of angiogenesis, suppression of tumor cell migration and invasion, and remodeling of the immune microenvironment. Due to its diverse modes of action, oridonin not only effectively inhibits melanoma cell proliferation and metastasis but also holds promise for synergistic effects when used in combination with current targeted therapies and immunotherapies.

In terms of clinical applications, while oridonin has shown promising anti-tumor activity and relatively manageable toxicity profiles in *in vitro* models, limitations such as poor water solubility, low stability, as well as the incomplete elucidation of potential resistance mechanisms and selectivity toward normal tissues remain critical barriers to its clinical translation. These limitations indicate that oridonin, in its current form, is not yet sufficient to replace existing standard therapies. Therefore, conducting further multicenter, high-quality clinical trials to systematically evaluate its pharmacokinetic properties, safety, and efficacy, as well as optimizing its delivery methods and dosage regimens, is key to advancing its clinical development. In particular, oridonin shows great potential for combination strategies, such as pairing with immune checkpoint inhibitors or BRAF inhibitors, which could enhance overall treatment efficacy for malignant melanoma while delaying the emergence of resistance. Furthermore, leveraging nanotechnology or chemical modifications to improve its bioavailability offers a promising approach to overcoming its current limitations in clinical applications.

In summary, oridonin exhibits definitive anti-tumor effects and significant research value in the field of malignant melanoma. Its multifaceted mechanisms of action provide a new therapeutic avenue for addressing the highly aggressive and drug-resistant nature of melanoma. With continued advancements in drug delivery systems and clinical studies, oridonin holds the potential to become an integral component of comprehensive melanoma treatment strategies in the future, offering patients more effective and safer therapeutic options.

## References

[1] Strashilov S Yordanov A. Aetiology and pathogenesis of cutaneous melanoma: current concepts and advances. Int J Mol Sci. (2021) 22:6395. doi: 10.3390/ijms22126395 34203771 PMC8232613

[2] Ahmed B Qadir MI Ghafoor S. Malignant melanoma: skin cancer-diagnosis, prevention, and treatment. Crit Rev Eukaryot Gene Expr. (2020) 30:291–7. doi: 10.1615/CritRevEukaryotGeneExpr.2020028454 32894659

[3] Pavri SN Clune J Ariyan S Narayan D. Malignant melanoma: beyond the basics. Plast Reconstr Surg. (2016) 138:330e–40e. doi: 10.1097/PRS.0000000000002367 27465194

[4] Lodde G Zimmer L Livingstone E SChadendorf D Ugurel S. Malignes melanom [Malignant melanoma. Pathologe. (2020) 41:281–92. doi: 10.1007/s00292-020-00776-x 32206873

[5] Kádár Z Lengyel Z Gyulai R. A melanoma Malignum sebészeti ellátása – Elvárható standardok [Surgery for Malignant melanoma - Expected standards. Magy Seb. (2023) 76:39–47. doi: 10.1556/1046.2023.10007 37130027

[6] Abbas O Miller DD Bhawan J. Cutaneous Malignant melanoma: update on diagnostic and prognostic biomarkers. Am J Dermatopathol. (2014) 36:363–79. doi: 10.1097/DAD.0b013e31828a2ec5 24803061

[7] Lazar AM Costea DO Popp CG Mastalier B. Skin Malignant melanoma and matrix metalloproteinases: promising links to efficient therapies. Int J Mol Sci. (2024) 25:7804. doi: 10.3390/ijms25147804 39063046 PMC11277423

[8] Liu X Xu J Zhou J Shen Q. Oridonin and its derivatives for cancer treatment and overcoming therapeutic resistance. Genes Dis. (2020) 8:448–62. doi: 10.1016/j.gendis.2020.06.010 PMC8209342 34179309

[9] Li X Zhang CT Ma W Xie X Huang Q. Oridonin: A review of its pharmacology, pharmacokinetics and toxicity. Front Pharmacol. (2021) 12:645824. doi: 10.3389/fphar.2021.645824 34295243 PMC8289702

[10] Ali MA Khan N Ali A Akram H Zafar N Imran K. Oridonin from Rabdosia rubescens: An emerging potential in cancer therapy - A comprehensive review. Food Sci Nutr. (2024) 12:3046–67. doi: 10.1002/fsn3.v12.5 PMC11077219 38726411

[11] Sobral PJM Vicente ATS Salvador JAR. Recent advances in oridonin derivatives with anticancer activity. Front Chem. (2023) 11:1066280. doi: 10.3389/fchem.2023.1066280 36846854 PMC9947293

[12] Niu Y Wang K Zhu X Zhang S Cherepanoff S Conway RM. The application of natural compounds in uveal melanoma drug discovery. J Pharm Pharmacol. (2022) 74:660–80. doi: 10.1093/jpp/rgac009 35532546

[13] Varghese R Dalvi YB. Natural products as anticancer agents. Curr Drug Targets. (2021) 22:1272–87. doi: 10.2174/1389450121999201230204526 33390130

[14] Naeem A Hu P Yang M Zhang J Liu Y Zhu W. Natural products as anticancer agents: current status and future perspectives. Molecules. (2022) 27:8367. doi: 10.3390/molecules27238367 36500466 PMC9737905

[15] Zhang Y Wang S Dai M Nai J Zhu L Sheng H. Solubility and bioavailability enhancement of oridonin: A review. Molecules. (2020) 25:332. doi: 10.3390/molecules25020332 31947574 PMC7024198

[16] Chopra A Sharma R Rao UNM. Pathology of melanoma. Surg Clin North Am. (2020) 100:43–59. doi: 10.1016/j.suc.2019.09.004 31753115

[17] Mao L Qi Z Zhang L Guo J Si L. Immunotherapy in acral and mucosal melanoma: current status and future directions. Front Immunol. (2021) 12:680407. doi: 10.3389/fimmu.2021.680407 34149718 PMC8212860

[18] Newell F Johansson PA Wilmott JS Nones K Lakis V Pritchard AL. Comparative genomics provides etiologic and biological insight into melanoma subtypes. Cancer Discov. (2022) 12:2856–79. doi: 10.1158/2159-8290.CD-22-0603 PMC9716259 36098958

[19] Bobos M. Histopathologic classification and prognostic factors of melanoma: a 2021 update. Ital J Dermatol Venerol. (2021) 156:300–21. doi: 10.23736/S2784-8671.21.06958-3 33982546

[20] Wang R Yan Q Liu X Wu J. Unraveling lipid metabolism reprogramming for overcoming drug resistance in melanoma. Biochem Pharmacol. (2024) 223:116122. doi: 10.1016/j.bcp.2024.116122 38467377

[21] Sapunar ZJ Pantoja AÁ Marín DL Ferrer-Rosende P. Epidemiología y características anatomoclínicas del melanoma Maligno en un instituto oncológico [Epidemiology and clinical features of Malignant melanoma. Analysis of 274 cases. Rev Med Chil. (2022) 150:1585–95. doi: 10.4067/s0034-98872022001201585 37906779

[22] Arnold M Singh D Laversanne M Vignat J Vaccarella S Meheus F. Global burden of cutaneous melanoma in 2020 and projections to 2040. JAMA Dermatol. (2022) 158:495–503. doi: 10.1001/jamadermatol.2022.0160 35353115 PMC8968696

[23] Tímár J Ladányi A. Molecular pathology of skin melanoma: epidemiology, differential diagnostics, prognosis and therapy prediction. Int J Mol Sci. (2022) 23:5384. doi: 10.3390/ijms23105384 35628196 PMC9140388

[24] Shah S Raskin L Cohan D Freeman M Hamid O. Treatment patterns of Malignant melanoma in the United States from 2011 to 2016: a retrospective cohort study. Curr Med Res Opin. (2020) 36:63–72. doi: 10.1080/03007995.2019.1662688 31469305

[25] Lauters R Brown AD Harrington KA. Melanoma: diagnosis and treatment. Am Fam Physician. (2024) 110:367–77. 39418569

[26] Villani A Scalvenzi M Micali G Lacarrubba F Fornaro L Martora F. Management of advanced invasive melanoma: new strategies. Adv Ther. (2023) 40:3381–94. doi: 10.1007/s12325-023-02555-5 PMC10329960 37306810

[27] Teimouri F Nikfar S Abdollahi M. Efficacy and side effects of dacarbazine in comparison with temozolomide in the treatment of Malignant melanoma: a meta-analysis consisting of 1314 patients. Melanoma Res. (2013) 23:381–9. doi: 10.1097/CMR.0b013e3283649a97 23880781

[28] Rydén V El-Naggar AI Koliadi A Ladjevardi CO Digkas E Valachis A. The role of dacarbazine and temozolomide therapy after treatment with immune checkpoint inhibitors in Malignant melanoma patients: A case series and meta-analysis. Pigment Cell Melanoma Res. (2024) 37:352–62. doi: 10.1111/pcmr.13156 38158376

[29] Strub T Ballotti R Bertolotto C. The “ART” of epigenetics in melanoma: from histone “Alterations, to resistance and therapies. Theranostics. (2020) 10:1777–97. doi: 10.7150/thno.36218 PMC6993228 32042336

[30] Long GV Swetter SM Menzies AM Gershenwald JE Scolyer RA. Cutaneous melanoma. Lancet. (2023) 402:485–502. doi: 10.1016/S0140-6736(23)00821-8 37499671

[31] Rashid S Shaughnessy M Tsao H. Melanoma classification and management in the era of molecular medicine. Dermatol Clin. (2023) 41:49–63. doi: 10.1016/j.det.2022.07.017 36410983

[32] Li C Kuai L Cui R Miao X. Melanogenesis and the targeted therapy of melanoma. Biomolecules. (2022) 12:1874. doi: 10.3390/biom12121874 36551302 PMC9775438

[33] Florent L Saby C Slimano F Morjani H. BRAF V600-mutated metastatic melanoma and targeted therapy resistance: an update of the current knowledge. Cancers (Basel). (2023) 15:2607. doi: 10.3390/cancers15092607 37174072 PMC10177463

[34] Anestopoulos I Kyriakou S Tragkola V Paraskevaidis I Tzika E Mitsiogianni M. Targeting the epigenome in Malignant melanoma: Facts, challenges and therapeutic promises. Pharmacol Ther. (2022) 240:108301. doi: 10.1016/j.pharmthera.2022.108301 36283453

[35] Ai L Xu A Xu J. Roles of PD-1/PD-L1 pathway: signaling, cancer, and beyond. Adv Exp Med Biol. (2020) 1248:33–59. doi: 10.1007/978-981-15-3266-5_3 32185706

[36] Snyder A Makarov V Merghoub T Yuan J Zaretsky JM Desrichard A. Genetic basis for clinical response to CTLA-4 blockade in melanoma. N Engl J Med. (2014) 371:2189–99. doi: 10.1056/NEJMoa1406498 PMC4315319 25409260

[37] Boutros C Tarhini A Routier E Lambotte O Ladurie FL Carbonnel F. Safety profiles of anti-CTLA-4 and anti-PD-1 antibodies alone and in combination. Nat Rev Clin Oncol. (2016) 13:473–86. doi: 10.1038/nrclinonc.2016.58 27141885

[38] Durante MA Rodriguez DA Kurtenbach S Kuznetsov JN Sanchez MI Decatur CL. Single-cell analysis reveals new evolutionary complexity in uveal melanoma. Nat Commun. (2020) 11:496. doi: 10.1038/s41467-019-14256-1 31980621 PMC6981133

[39] Ott PA Hu-Lieskovan S Chmielowski B Govindan R Naing A Bhardwaj N. A phase ib trial of personalized neoantigen therapy plus anti-PD-1 in patients with advanced melanoma, non-small cell lung cancer, or bladder cancer. Cell. (2020) 183:347–362.e24. doi: 10.1016/j.cell.2020.08.053 33064988

[40] Czarnecka AM Bartnik E Fiedorowicz M Rutkowski P. Targeted therapy in melanoma and mechanisms of resistance. Int J Mol Sci. (2020) 21:4576. doi: 10.3390/ijms21134576 32605090 PMC7369697

[41] Wang DR Wu XL Sun YL. Therapeutic targets and biomarkers of tumor immunotherapy: response versus non-response. Signal Transduct Target Ther. (2022) 7:331. doi: 10.1038/s41392-022-01136-2 36123348 PMC9485144

[42] Natarelli N Aleman SJ Mark IM Tran JT Kwak S Botto E. A review of current and pipeline drugs for treatment of melanoma. Pharm (Basel). (2024) 17:214. doi: 10.3390/ph17020214 PMC10892880 38399429

[43] Wong SK Nebhan CA Johnson DB. Impact of patient age on clinical efficacy and toxicity of checkpoint inhibitor therapy. Front Immunol. (2021) 12:786046. doi: 10.3389/fimmu.2021.786046 34868071 PMC8635107

[44] Soltantoyeh T Akbari B Karimi A Mahmoodi Chalbatani G Ghahri-Saremi N Hadjati J. Chimeric antigen receptor (CAR) T cell therapy for metastatic melanoma: challenges and road ahead. Cells. (2021) 10:1450. doi: 10.3390/cells10061450 34207884 PMC8230324

[45] Raaijmakers MI Rozati S Goldinger SM Widmer DS Dummer R Levesque MP. Melanoma immunotherapy: historical precedents, recent successes and future prospects. Immunotherapy. (2013) 5:169–82. doi: 10.2217/imt.12.162 23413908

[46] Serratì S Guida M Di Fonte R De Summa S Strippoli S Iacobazzi RM. Circulating extracellular vesicles expressing PD1 and PD-L1 predict response and mediate resistance to checkpoint inhibitors immunotherapy in metastatic melanoma. Mol Cancer. (2022) 21:20. doi: 10.1186/s12943-021-01490-9 35042524 PMC8764806

[47] Tirosh I Izar B Prakadan SM Wadsworth MH 2nd Treacy D Trombetta JJ. Dissecting the multicellular ecosystem of metastatic melanoma by single-cell RNA-seq. Science. (2016) 352:189–96. doi: 10.1126/science.aad0501 PMC4944528 27124452

[48] Malissen N Grob JJ. Treatment of recurrent melanoma following adjuvant therapy. Am J Clin Dermatol. (2023) 24:333–41. doi: 10.1007/s40257-023-00762-y 36890427

[49] Žiaran M Dvořák P Hoffmann P Kopecký J. Failure of adjuvant treatment for Malignant melanoma - what next? Klin Onkol. (2021) 34:73–7. doi: 10.48095/ccko202173 33657824

[50] Allen KJH Malo ME Jiao R Dadachova E. Targeting melanin in melanoma with radionuclide therapy. Int J Mol Sci. (2022) 23:9520. doi: 10.3390/ijms23179520 36076924 PMC9455397

[51] Li Z Gao Y Cao Y He F Jiang R Liu H. Extracellular RNA in melanoma: Advances, challenges, and opportunities. Front Cell Dev Biol. (2023) 11:1141543. doi: 10.3389/fcell.2023.1141543 37215082 PMC10192583

[52] Feng Q Xu X Zhang S. Nrf2 protein in melanoma progression, as a new means of treatment. Pigment Cell Melanoma Res. (2024) 37:247–58. doi: 10.1111/pcmr.13137 37777339

[53] Shi H Cheng Z. MC1R and melanin-based molecular probes for theranostic of melanoma and beyond. Acta Pharmacol Sin. (2022) 43:3034–44. doi: 10.1038/s41401-022-00970-y PMC9712491 36008707

[54] Tang M Duan T Lu Y Liu J Gao C Wang R. Tyrosinase-woven melanin nets for melanoma therapy through targeted mitochondrial tethering and enhanced photothermal treatment. Adv Mater. (2024) 36:e2411906. doi: 10.1002/adma.202411906 39285827

[55] Guo M Fang W Hu Z. Traditional Chinese medicine and its components effectively reduce resistance mediated by immune checkpoint inhibitors. Front Immunol. (2024) 15:1429483. doi: 10.3389/fimmu.2024.1429483 39660124 PMC11628391

[56] Shang Y Zhao H. Research progress of Chinese medicinal monomers in the process of melanoma occurrence. Pharm Biol. (2025) 63:53–67. doi: 10.1080/13880209.2024.2445695 39790031 PMC11727062

[57] Lai Y Feng Q Zhang R Shang J Zhong H. The great capacity on promoting melanogenesis of three compatible components in vernonia anthelmintica (L.) willd. Int J Mol Sci. (2021) 22:4073. doi: 10.3390/ijms22084073 33920793 PMC8071200

[58] Wang K Chen Q Shao Y Yin S Liu C Liu Y. Anticancer activities of TCM and their active components against tumor metastasis. BioMed Pharmacother. (2021) 133:111044. doi: 10.1016/j.biopha.2020.111044 33378952

[59] Zhao Y Yu S Wang Y Chen Y Chen J Wang J. Pueraria protein extract inhibits melanogenesis and promotes melanoma cell apoptosis through the regulation of MITF and mitochondrial−related pathways. Mol Med Rep. (2023) 27:64. doi: 10.3892/mmr.2023.12951 36734267 PMC9926868

[60] Yang Q Ma W Yu K Zhang Q Ye Z Xia W. Oridonin suppresses human gastric cancer growth *in vitro* and *in vivo* via inhibition of VEGF, integrin β3, and PCNA. Biol Pharm Bull. (2020) 43:1035–45. doi: 10.1248/bpb.b19-00839 32612067

[61] Li D Han T Liao J Hu X Xu S Tian K. Oridonin, a promising ent-kaurane diterpenoid lead compound. Int J Mol Sci. (2016) 17:1395. doi: 10.3390/ijms17091395 27563888 PMC5037675

[62] National Center for Biotechnology Information. PubChem compound summary for CID 5321010, Oridonin. Retrieved February 17, 2025 (2025). Available online at: https://pubchem.ncbi.nlm.nih.gov/compound/Oridonine.

[63] Xu J Wold EA Ding Y Shen Q Zhou J. Therapeutic potential of oridonin and its analogs: from anticancer and antiinflammation to neuroprotection. Molecules. (2018) 23:474. doi: 10.3390/molecules23020474 29470395 PMC6017549

[64] Lin FE Zhang XY Zhang YP Wang J. Preparation, characterization, and pharmacokinetics of oridonin-loaded liposomes. BioMed Chromatogr. (2023) 37:e5603. doi: 10.1002/bmc.v37.5 36781382

[65] Islam MT Bardaweel SK Mubarak MS Koch W Gaweł-Beben K Antosiewicz B. Immunomodulatory effects of diterpenes and their derivatives through NLRP3 inflammasome pathway: A review. Front Immunol. (2020) 11:572136. doi: 10.3389/fimmu.2020.572136 33101293 PMC7546345

[66] Li DH Hu P Xu ST Fang CY Tang S Wang XY. Lasiokaurin derivatives: synthesis, antimicrobial and antitumor biological evaluation, and apoptosis-inducing effects. Arch Pharm Res. (2017) 40:796–806. doi: 10.1007/s12272-016-0867-9 28110416

[67] Zhang M Hou L Tang W Lei W Lin H Wang Y. Oridonin attenuates atherosclerosis by inhibiting foam macrophage formation and inflammation through FABP4/PPARγ signalling. J Cell Mol Med. (2023) 27:4155–70. doi: 10.1111/jcmm.v27.24 PMC10746953 37905351

[68] Ding Y Ding C Ye N Liu Z Wold EA Chen H. Discovery and development of natural product oridonin-inspired anticancer agents. Eur J Med Chem. (2016) 122:102–17. doi: 10.1016/j.ejmech.2016.06.015 PMC5003635 27344488

[69] Cheng W Huang C Ma W Tian X Zhang X. Recent development of oridonin derivatives with diverse pharmacological activities. Mini Rev Med Chem. (2019) 19:114–24. doi: 10.2174/1389557517666170417170609 28425866

[70] Zhang CL Wu LJ Tashiro S Onodera S Ikejima T. Oridonin induced A375-S2 cell apoptosis via bax-regulated caspase pathway activation, dependent on the cytochrome c/caspase-9 apoptosome. J Asian Nat Prod Res. (2004) 6:127–38. doi: 10.1080/1028602031000147375 15008459

[71] Wang HJ Li D Yang FY Tashiro S Onodera S Ikejima T. Oridonin induces human melanoma A375-S2 cell death partially through inhibiting insulin-like growth factor 1 receptor signaling. J Asian Nat Prod Res. (2008) 10:787–98. doi: 10.1080/10286020802030918 18696333

[72] Zhang CL Wu LJ Zuo HJ Tashiro S Onodera S Ikejima T. Cytochrome c release from oridonin-treated apoptotic A375-S2 cells is dependent on p53 and extracellular signal-regulated kinase activation. J Pharmacol Sci. (2004) 96:155–63. doi: 10.1254/jphs.FPJ04008X 15492467

[73] Abdullah NA Md Hashim NF Ammar A Muhamad Zakuan N. An insight into the anti-angiogenic and anti-metastatic effects of oridonin: current knowledge and future potential. Molecules. (2021) 26:775. doi: 10.3390/molecules26040775 33546106 PMC7913218

[74] Jiang JH Pi J Cai JY. Oridonin exhibits anti-angiogenic activity in human umbilical vein endothelial cells by inhibiting VEGF-induced VEGFR-2 signaling pathway. Pathol Res Pract. (2020) 216:153031. doi: 10.1016/j.prp.2020.153031 32703495

[75] Li CY Wang Q Shen S Wei XL Li GX. Oridonin inhibits migration, invasion, adhesion and TGF-β1-induced epithelial-mesenchymal transition of melanoma cells by inhibiting the activity of PI3K/Akt/GSK-3β signaling pathway. Oncol Lett. (2018) 15:1362–72. doi: 10.3892/ol.2017.7421 PMC5772702 29399187

[76] Geertsen R Hofbauer G Kamarashev J Yue FY Dummer R. Immune escape mechanisms in Malignant melanoma. Int J Mol Med. (1999) 3:49–57. doi: 10.3892/ijmm.3.1.49 9864385

[77] Liu S Wang X Sun X Wei B Jiang Z Ouyang Y. Oridonin inhibits bladder cancer survival and immune escape by covalently targeting HK1. Phytomedicine. (2024) 126:155426. doi: 10.1016/j.phymed.2024.155426 38367425

[78] Guo J Chen T Ma Z Qiao C Yuan F Guo X. Oridonin inhibits 4T1 tumor growth by suppressing Treg differentiation via TGF-β receptor. Int Immunopharmacol. (2020) 88:106831. doi: 10.1016/j.intimp.2020.106831 32853925

[79] Hwang TL Chang CH. Oridonin enhances cytotoxic activity of natural killer cells against lung cancer. Int Immunopharmacol. (2023) :122:110669. doi: 10.1016/j.intimp.2023.110669 37480753

[80] Liu YF Jia Y He PC Zhang M He Q. Influence of oridonin on the icilling acitivity of NK-92 MI cells targeting cell THP1 and its mechanism. Zhongguo Shi Yan Xue Ye Xue Za Zhi. (2019) 27:1374–9. doi: 10.19746/j.cnki.issn.1009-2137.2019.05.004 31607286

[81] Jin K-T Lu Z-B Chen J-Y. Recent trends in nanocarrier-based targeted chemotherapy: selective delivery of anticancer drugs for effective lung, colon, cervical, and breast cancer treatment. J Nanomater. (2020) 2020:9184284. doi: 10.1155/2020/9184284

[82] Yang Z Huang S Liu Y Chang X Liang Y Li X. Biotin-targeted au(I) radiosensitizer for cancer synergistic therapy by intervening with redox homeostasis and inducing ferroptosis. J Med Chem. (2022) 65:8401–15. doi: 10.1021/acs.jmedchem.2c00300 35687871

[83] Bakalova R Semkova S Ivanova D Zhelev Z Miller T Takeshima T. Selective targeting of cancerous mitochondria and suppression of tumor growth using redox-active treatment adjuvant. Oxid Med Cell Longev. (2020) 2020:6212935. doi: 10.1155/2020/6212935 33204397 PMC7652615

[84] Zhang C Chen Y Zuo Y Wang M Chen H Wang C. Dual targeting of FR+CD44 overexpressing tumors by self-assembled nanoparticles quantitatively conjugating folic acid-hyaluronic acid to the GSH-sensitively modified podophyllotoxin. Chem Eng J. (2025) 505:159276. doi: 10.1016/j.cej.2025.159276

[85] Chen Y Yang J Wang C Wang T Zeng Y Li X. Aptamer-functionalized triptolide with release controllability as a promising targeted therapy against triple-negative breast cancer. J Exp Clin Cancer Res. (2024) 43:207. doi: 10.1186/s13046-024-03133-5 39054545 PMC11270970

[86] Patel P Garala K Singh S Prajapati BG Chittasupho C. Lipid-based nanoparticles in delivering bioactive compounds for improving therapeutic efficacy. Pharm (Basel). (2024) 17:329. doi: 10.3390/ph17030329 PMC10975431 38543115

[87] Shen J Gao F Pan Q Zong Z Liang L. Synthesis and application of a pH-responsive functional metal-organic framework: *in vitro* investigation for delivery of oridonin in cancer therapy. Molecules. (2024) 29:2643. doi: 10.3390/molecules29112643 38893518 PMC11173415

[88] Wang C Xu H Chen Y Li X Chen H Liu J. Hydroxyl-based acid-hypersensitive acetal ester bond: Design, synthesis and the application potential in nanodrugs. Eur J Med Chem. (2025) 283:117153. doi: 10.1016/j.ejmech.2024.117153 39681042

[89] Guo J Huang M Hou S Yuan J Chang X Gao S. Therapeutic potential of terpenoids in cancer treatment: targeting mitochondrial pathways. Cancer Rep (Hoboken). (2024) 7:e70006. doi: 10.1002/cnr2.70006 39234662 PMC11375335

[90] Basu R Baumgaertel N Wu S Kopchick JJ. Growth hormone receptor knockdown sensitizes human melanoma cells to chemotherapy by attenuating expression of ABC drug efflux pumps. Horm Cancer. (2017) 8:143–56. doi: 10.1007/s12672-017-0292-7 PMC10355985 28293855

[91] Kreidieh FY Tawbi HA. The introduction of LAG-3 checkpoint blockade in melanoma: immunotherapy landscape beyond PD-1 and CTLA-4 inhibition. Ther Adv Med Oncol. (2023) 15:17588359231186027. doi: 10.1177/17588359231186027 37484526 PMC10357068

[92] Rascio F Spadaccino F Rocchetti MT Castellano G Stallone G Netti GS. The pathogenic role of PI3K/AKT pathway in cancer onset and drug resistance: an updated review. Cancers (Basel). (2021) 13:3949. doi: 10.3390/cancers13163949 34439105 PMC8394096

[93] Jin P Jiang J Zhou L Huang Z Nice EC Huang C. Mitochondrial adaptation in cancer drug resistance: prevalence, mechanisms, and management. J Hematol Oncol. (2022) 15:97. doi: 10.1186/s13045-022-01313-4 35851420 PMC9290242

[94] Zheng Q Zhang M Zhou F Zhang L Meng X. The breast cancer stem cells traits and drug resistance. Front Pharmacol. (2021) 11:599965. doi: 10.3389/fphar.2020.599965 33584277 PMC7876385

[95] Haus-Cohen M Reiter Y. Harnessing antibody-mediated recognition of the intracellular proteome with T cell receptor-like specificity. Front Immunol. (2024) 15:1486721. doi: 10.3389/fimmu.2024.1486721 39650646 PMC11621052

